# Journal of Clinical Monitoring and Computing 2015 end of year summary: tissue oxygenation and microcirculation

**DOI:** 10.1007/s10877-016-9846-4

**Published:** 2016-02-20

**Authors:** T. W. L. Scheeren

**Affiliations:** Department of Anesthesiology, University Medical Center Groningen, University of Groningen, Groningen, Netherlands

**Keywords:** Monitoring, Tissue oxygenation, Microcirculation, NIRS, Vascular occlusion test, Mitochondrial function

## Abstract

Last year we started this series of end of year summaries of papers published in the 2014 issues of the Journal Of Clinical Monitoring And Computing with a review on near infrared spectroscopy (Scheeren et al. in J Clin Monit Comput 29(2):217–220, 2015). This year we will broaden the scope and include papers published in the field of tissue oxygenation and microcirculation, or a combination of both entities. We present some promising new technologies that might enable a deeper insight into the (patho)physiology of certain diseases such as sepsis, but also in healthy volunteers. These may help researchers and clinicians to evaluate both tissue oxygenation and microcirculation beyond macro-hemodynamic measurements usually available at the bedside.

## Monitoring tissue oxygenation

Ensuring an adequate tissue oxygenation may be considered the “holy grail” of cardiovascular management, and all variables we routinely measure in the perioperative and critical care setting (stroke volume, heart rate, cardiac output, Hb, S_a_O_2_, p_a_O_2_, DO_2_, etc.) contribute to this ultimate goal. Tissue oxygenation is the oxygen tension measured at the capillary level, the interstitial space and even intracellular in the mitochondria, and it gives information about the balance between local oxygen delivery (DO_2_) and oxygen consumption (**V**O_2_) [1]. While the oxygen tension within an individual organ remains reasonably comparable across species, there are intra-organ differences, depending on regional variations in organ flow and activity [2].

There are quite a number of methodologies available for estimating or measuring tissue oxygenation at the different levels, such as Clark type electrodes, near infrared spectroscopy (NIRS), reflectance spectroscopy, and mitochondrial pO_2_ measurements. The most popular of these is certainly NIRS [3], a fact that is reflected by the number of papers published last year in the JCMC.

In the August issue, Koch et al. [4] presented an interesting prospective observational study in which they looked at tissue oxygenation at two sites (forehead and thenar muscle) using NIRS technology in 50 patients with severe sepsis or septic shock. These regional oxygenation values (rSO_2_) were compared to central venous oxygen saturation (ScvO_2_), which often serves as a treatment goal for optimizing tissue oxygenation in these patients [5]. In particular, the authors wanted to know if central (ScvO_2_) and regional (rSO_2_) indicators of tissue oxygenation are correlated and if they could be used to predict outcome in septic patients. They found that mean baseline rSO_2_ in their septic patients was 60 ± 11 %, which was considerably lower than that observed in non-septic patients undergoing major surgery (86 ± 6 %) [6] or in non-septic ICU patients (80 ± 7 %) [7]. There was a moderate correlation between frontal and thenar rSO_2_, however, no relation to outcome (i.e. similar values between survivors and non-survivors with a hospital mortality of 52 %). Similarly, there was a weak correlation between thenar rSO_2_ and ScvO_2_, as also described previously [6]. Nevertheless, frontal rSO_2_ was able to predict a ScvO_2_ below 70 %. Surprisingly, ScvO_2_ was higher in non-survivors (76 ± 8 %) than in survivors (71 ± 8 %) at baseline, suggesting an oxygen extraction or utilization problem in the first group. As such, a high baseline ScvO_2_ (>70 %) but not rSO_2_ was predictive of mortality, particularly when combined with a lactate level >2.5 mmol/l. This interesting finding raises the question if targeting a ScvO_2_ > 70 % (as described in current guidelines such as the surviving sepsis campaign) [8] is really beneficial for the patient with sepsis. Unfortunately, the authors did not perform a vascular occlusion test (VOT) in their patients, which would have given further information on muscle metabolism (**V**O_2_, derived from the descending slope) and of (micro)vascular reperfusion (vasoreactivity, derived from the ascending slope) (see below). Both slopes were related to outcome, with non-survivors having increased descending slopes [9] and lower reperfusion slopes [7].

In the accompanying editorial comment to this paper, Mesquida points out that regional rSO_2_ and global ScvO_2_ are not reflecting exactly the same entity, so that the usefulness of predicting the one by the other may be questionable [10]. Yet, unlike ScvO_2_ measurements, rSO_2_ measurements are purely non-invasive and give an instantaneous result, which qualifies NIRS as a screening tool for detecting tissue hypoxia early in the resuscitation process. Instead of using rSO_2_ measurements to predict ScvO_2_ measurements, Mesquida suggests an integrative approach, combining global and regional markers of tissue oxygenation to detect tissue dysoxia at the bedside [10]. Furthermore, Mesquida evaluates the association between ScvO_2_ and lactate with mortality by describing four scenarios: (1) adequate global DO_2_ and cellular metabolism (normal lactate and normal ScvO_2_); (2) oxygen deficit (normal lactate and low ScvO_2_); (3) oxygen debt (high lactate and low ScvO_2_); and (4) impaired oxygen extraction (high lactate and high ScvO_2_). The latter scenario was associated with the highest mortality (80 %) in the Koch study [4], although an inadequate DO_2_ was probably not the underlying problem. This important finding raises doubts on the benefit of the current clinical practice of maximizing DO_2_ during the resuscitation of septic patients.

In the August issue, Murphy and colleagues evaluated the utility of cerebral oxygenation (ScO_2_) measurements obtained by NIRS in a small group of patients undergoing hepato-biliary surgery [11]. In these patients, increased bilirubin concentrations can interfere with the NIRS measurements by absorbing infrared light at similar wavelengths as deoxygenated hemoglobin, making the obtained ScO_2_ values unreliably low. In view of the well-known fact that changes in ScO_2_ values from individual baseline are more important than absolute ScO_2_ values, the authors investigated if the time course of ScO_2_ values is affected in patients with high bilirubin levels, other than starting from a lower baseline value. Patients were divided into two groups using a ScO_2_ cut-off value of 51 %, which has been identified as threshold value for detecting cerebral ischemia [12]. They found that although starting from different baseline levels, the reactions to preoxygenation as well as the intraoperative time course of ScO_2_ values were similar between groups. Therefore, the authors conclude that trends in cerebral oximetry may also be useful in patients with liver dysfunction and increased bilirubin levels, even if absolute ScO_2_ values are falsely low and thus do not *per se* indicate cerebral ischemia.

In the same issue, Ritzenthaler et al. [13] report on a small case series of ScO_2_ variation in three patients undergoing mechanical thrombectomy after acute ischemic stroke. As expected, baseline ScO_2_ was lower in the infarcted compared to the non-infarcted side, resulting in an interhemispheric difference >5 %. After successful recanalization (which occurred in two of the three patients), ScO_2_ at the infarcted side increased by about 10 %, resulting in a decrease of the interhemispheric difference. In contrast, in the patient where the thrombectomy failed, ScO_2_ values remained constant. The authors conclude that cerebral NIRS monitoring could be used to monitor the success of recanalization therapy of acute ischemic stroke, and that the continuous ScO_2_ readings might provide additional information to instantaneous CT or MRI imaging. It has to be noted, however, that ScO_2_ measurements as usually performed at the forehead typically assess a sample volume of the watershed area between the anterior and middle cerebral artery territory, and may not detect ischemic events in other regions of the brain, particularly those in the posterior region.

A more traditional approach is the monitoring of ScO_2_ during carotid endarterectomy (CEA). In a prospective observational study involving 69 patients undergoing CEA, Perez et al. [14] followed changes in ScO_2_ as well as processed EEG responses to carotid clamping and shunting. In general, shunting of the clamped carotid artery is performed in about a quart of all CEA cases, and, in view of the potential complications associated with the shunting procedure, there is not enough evidence to support or refute its use as of yet. Detection of cerebral ischemia by monitoring ScO_2_ or processed EEG might help identify patients who benefit from carotid shunting when this procedure is performed under general anesthesia. The authors measured bilateral BIS (bispectral index) and ScO_2_ and found that after clamping ScO_2_ at the surgical-side was significantly lower than at the contralateral side until the shunt was removed and blood flow was restored in the repaired carotid artery. BIS decreased bilaterally to a value significantly below baseline following clamping but returned to a value near baseline following shunt activation with no differences between sides. In other words: while ScO_2_ measurements could clearly differentiate between surgical and contralateral side, BIS (although measured bilaterally) could not. Furthermore, the minimum ScO_2_ and BIS values observed after carotid clamping did not correlate with the degree of carotid stenosis determined preoperatively. A nice side-effect discussed by the authors relates to the discussion on “extracranial contamination” of the NIRS signal; [15] during CEA, the common, internal and external carotid arteries are clamped prior to insertion of the shunt. After the shunt is placed, the common and internal carotid arteries are unclamped, while the external carotid artery remains clamped, resulting in decreased perfusion of superficial and extracranial tissues. The study results clearly show that ScO_2_ increases after activation of the shunt due to actual changes in intracranial blood flow. Important limitations of the study include the lack of blood pressure control and, due to the pure observational character, the fact that no conclusions can be drawn on the possible impact of either monitoring on neurological outcome. Finally, a cost-benefit analysis should be performed to evaluate if a reduction in neurological complications justifies the additional costs of the monitoring.

In the same issue, Eichhorn and colleagues report on the ScO_2_ response during hypoxia in apnea divers [16]. What at first view looks like a pure physiological study with little clinical applicability, is actually of high relevance to the clinical setting of anesthesia: the authors intended to mimic dynamic hypoxic situations such as those occurring during difficult intubation, “cannot ventilate cannot intubate” situations or laryngospasm, as well as during cardiopulmonary resuscitation (CPR). For this purpose, they asked 10 experienced apneic divers to perform a maximal breath hold manoeuvre. They found that during apnea both peripheral oxygen saturation (SpO_2_) and ScO_2_ significantly decreased with a close correlation of both variables after normalization to individual baseline values. More importantly, ScO_2_ significantly faster returned to baseline values after the end of apnea than SpO_2_, suggesting that cerebral oximetry might be useful under conditions of airway problems and in pulseless CPR situations where SpO_2_ monitoring fails. In a second set of experiments, the authors looked also at peripheral tissue oxygenation as measured via NIRS above the quadriceps muscle, and found that these measurements decreased even earlier than SpO_2_ and ScO_2_ values during apnea, probably as a result of peripheral vasoconstriction. This theory is supported by the finding that the hemoglobin volume (measured also with NIRS) decreased in the peripheral muscle, whereas it remained constant in the brain, probably due to cerebral blood flow autoregulation.

In an animal study, Kurita et al. [17] investigated the effects of beta-blockade and hemodilution on ScO_2_. It is known that during acute hemodilution beta-blockers reduce ScO_2_, probably by blunting the cardiac response to hemodilution. This has clinical implications, for instance in situations where controlled hypotension is induced with beta-blockers and surgical bleeding occurs. Using anesthetized pigs allowed the authors to systematically combine different beta-blocker dosages and hemodilution conditions, and in addition looking at the impact of cardio-selectivity on ScO_2_ by using two different short-acting beta-blockers, one more cardio-selective (landiolol) than the other (esmolol). While both beta-blockers had no effect on ScO_2_ before hemodilution, they decreased ScO_2_ dose-dependently at the different stages of hemodilution with no difference between drugs. While the effects of the cardio-selective landiolol on ScO_2_ were reversible after stopping drug infusion, ScO_2_ remained below preinfusion values after stopping esmolol during hemodilution. Of note, hemodilution up to 66 % *per se* did not reduce ScO_2_ in this animal model, since cardiac output compensatorily increased by about 50 % to maintain DO_2_. When these experimental findings were transferred to the clinical setting, they imply that perioperative ScO_2_ monitoring might be particularly useful in patients receiving beta-blockers and undergoing surgery where major bleeding might occur. Furthermore, it should be investigated further if cardio-selective beta-blockers impair cerebral oxygenation to a lesser extent under these conditions than unselective beta-blockers.

## Combined monitoring of microcirculation and tissue oxygenation

A hot topic in perioperative medicine and intensive care since several years is the monitoring of fluid responsiveness. Klijn et al. [18] tried to evaluate how monitoring of the microcirculation (tissue perfusion) and tissue oxygenation can be used for this purpose. In a cohort of 35 critically ill patients with sepsis, they measured sublingual microcirculatory perfusion with side-stream dark field (SDF) imaging and also skin perfusion and oxygenation by combined laser Doppler and reflectance spectroscopy, a diagnostic tool for assessing microvascular blood flow and tissue oxygen consumption at the bedside [19]. By defining fluid responsiveness as an increase in stroke volume (SV) by >5 % in response to a 250 ml colloid infusion, the authors found that in fluid responders, all markers of microcirculatory perfusion and tissue oxygenation increased, despite the fact that initial fluid resuscitation had been performed before starting the current fluid bolus. They conclude that their non-invasive methods to assess the microcirculation and tissue oxygenation were not inferior to the traditional, invasive hemodynamic measurements usually performed to monitor fluid responsiveness in clinical practice, and could therefore help guiding fluid therapy particularly in situations where the risk of harmful fluid overloading is increased (such as in sepsis). The fact that the changes in microcirculatory flow and tissue oxygenation were relatively low (albeit significant) might be explained by the facts that 1) the fluid challenge was performed after initial volume resuscitation and 2) that the sublingual microcirculation was already “hyperdynamic” before the fluid challenge, a finding typical for the later stage of sepsis [20]. Finally, the techniques used by the authors do not belong to the current clinical armamentarium and are rather research tools as of yet, mainly due to the necessity for off-line analysis and substantial intra- and interrater variability. Future developments may help bringing these techniques to the bedside, allowing for routine measurements and, most importantly, for collecting data showing an impact on patient outcome.

As mentioned above, the vascular occlusion test in combination with NIRS monitoring at the upper or lower extremity can reveal valuable information on the reaction of the microcirculation. After occlusion of the vessels supplying blood flow to the extremity where tissue oxygenation is measured by inflating a cuff above systolic blood pressure, the descending or desaturation slope gives information about the oxygen metabolism (**V**O_2_) of the muscle under the NIRS probe. After deflation of the cuff following limb ischemia, the upstroke or reperfusion slope of the tissue oxygenation curve can be taken as a measure of microvascular reactivity and recruitment, i.e. the ability to washout the stagnant deoxygenated blood from the measurement volume and replace it by “fresh”, oxygenated blood [21–23].

Two studies published in the JCMC last year used the VOT in combination with peripheral NIRS measurements [23, 24]. In the first study, Zamparini et al. [24] examined if the VOT could be used to differentiate NIRS-derived vascular reactivity between smokers and non-smokers. For this purpose, they included 20 healthy volunteers of both sexes (10 smokers and 10 non-smokers) and hypothesized that endothelial dysfunction usually seen in smokers could be detected by revealing microcirculatory dysfunction using NIRS and VOT above a leg muscle. However, there were absolutely no differences between smokers and non-smokers, neither in desaturation slope during ischemia nor in resaturation rates during reperfusion. Possible explanations provided by the authors include the inability or lack of sensitivity of NIRS measurements to detect smoking-induced endothelial dysfunction on the one hand and a “contamination” of the NIRS-derived hemoglobin oxygen saturation measurements by muscular myoglobin on the other. Furthermore, the choice of otherwise healthy, young volunteers (mean age 20 years) with a relatively short smoking history (10 pack-years) might have contributed to the negative findings.

In the second study on NIRS VOT published in the April issue, Lee et al. investigated if the NIRS device used matters when a VOT is performed. As the VOT has initially been described using the InSpectra^®^ device (Hutchinson Technology, USA) and thenar rSO_2_ measurements, the authored wondered if other NIRS devices (primarily designed for measuring ScO_2_) could be used interchangeably for this purpose. In 20 healthy volunteers they compared rSO_2_ measurements derived from two different devices (InSpectra^®^ and INVOS^®^, Covidien) during a VOT in the same subject. While the baseline values were significantly lower with the INVOS^®^ as compared to those of the InSpectra^®^ (75 vs. 82 %), the minimum rSO_2_ during VOT was reached earlier with the INVOS^®^. Similarly, the desaturation and reoxygenation rates were higher as measured with the INVOS^®^. These results show that either device can be used for measuring rSO_2_ during a VOT, a finding that has been reported earlier [12, 25], but that device-specific differences in baseline values and dynamic changes should be considered.

## Monitoring the microcirculation

The microcirculation is a central part of the cardiovascular system consisting of blood vessels with a diameter of <150 µm. The main function of the microcirculation is to deliver oxygen (DO_2_) to the cells and maintain tissue oxygenation. The normal microcirculation is characterized by a dense network of perfused capillaries with minimal heterogeneity, most of the capillaries being perfused even though flow in the various capillaries varies according to metabolic needs of the surrounding tissues. Adaptation to metabolic needs occurs by opening and closing capillaries, and adapting the velocity of circulating cells within these. Modulation of precapillary sphincters is partly under the influence of systemic factors, with sympathetic stimulation and circulating substances, whereas the fine-tuning perfusion is regulated by local factors that include direct stimulation of endothelial cells by backward communication and local release of nitric oxide by red blood cells under hypoxic conditions.

In recent years, a large body of knowledge supports a central pathophysiological importance of the microcirculation in the development of organ failure. Microcirculatory alterations are more severe in non-survivors than in survivors. Improvements in microvascular perfusion occurring in response to goal resuscitation therapies are associated with subsequent improvements in organ function while organ function further deteriorated when microvascular perfusion failed to improve or even worsened. Thus, the state of the microcirculation might be considered an important prognostic indicator.

For many years, the direct study of the microcirculation was restricted to animal experimentation. The recent introduction of video microscopy using hand-held microscopes allows the non-invasive visualization of the microcirculation in patients. The analysis of the microcirculation should include parameters of vessel density (total and perfused vascular densities), perfusion [proportion of perfused vessels (PPVs) and microvascular flow index (MFI)], and heterogeneity [26]. Particularly, the sublingual region has been frequently assessed for video microscopy due to its good accessibility and its close correlation with other vascular beds, e.g. the intestinal perfusion. Recently, a new device based on incident dark-field (IDF) imaging (Cytocam) was developed for this application, with improved optical lenses, a high-resolution computer controlled image sensor, and an application for automatic analysis [27]. In a study on preterm neonates, van Elteren and colleagues compared this new IDF imaging technique to the older SDF imaging method [28]. Neonates were chosen since their reduced skin thickness facilitates transcutaneous non-invasive measurements on the inner upper arm. The authors found that IDF imaging visualized 20 % more vessels (particularly small vessels) than the older SDF device, resulting in a higher observed vessel density and a lower perfusion (as determined by the proportion of perfused vessels, PPV). When the authors looked at the imaging quality, the IDF images scored better than the SDF video images (25 % more optimal scores using the standardized imaging quality score) [29]. This may be due to technical advancement in the image acquisition as well as in the lower weight of the handheld camera (120 vs. 350 g), allowing a reduction of motion and pressure artefacts.

In an accompanying editorial comment, Lehmann and colleagues point out that both cameras (i.e. IDF and SDF) have a different optical resolution and a distinctive size of observation area, so that these different microcirculatory windows should affect the comparison, even if the same off-line analysis software was used [30]. They also expect that the introduction of automated analysis software packages will initiate more validation studies as a prerequisite to the introduction of these promising technologies into clinical practice.

Another step forward is made by the group of Harms et al., who extended their microcirculatory approach by looking “even further down the road” into the cell, more precisely into mitochondrial function [31]. The authors used a technique that was developed earlier by this group, namely the so-called Protoporphyrin IX—Triplet State Lifetime Technique. This technique, the details of which cannot be comprehensively reproduced in this review, provides a new approach for measuring mitochondrial function and oxygen tension (mitoPO_2_) in vivo. In the current paper, the authors describe a modification of this invasive technique for transcutaneous application. Furthermore, they combined the technique with what they call oxygen disappearance rate (ODR) measurements, i.e. measuring the decline in mitoPO_2_ after pressure-induced cessation of blood supply (and thus DO_2_). From these ODR measurements they derive values of mitochondrial oxygen consumption (mito**V**O_2_). In their paper, the authors present two sets of experiments, an animal study in rats (with or without lipopolysaccharide, LPS) and a volunteer study.

In the rats, the authors determined mitoPO_2_ and mito**V**O_2_ in different organs (skin, liver and buccal mucosa) and compared this to skin perfusion and oxygenation as assessed by combined laser Doppler and reflectance spectroscopy [19]. While absolute values for skin oxygenation differed from those of the other organs, mito**V**O_2_ (but not mitoPO_2_) decreased in all organs after LPS. Nevertheless, the authors conclude from this that the skin can be used as a substitute for other less accessible target organs to monitor alterations in mitochondrial respiration in critical illness like sepsis and septic shock. Although mitoPO_2_ represents the lowest end of the oxygen distribution in tissues [2], the values found by the authors were surprisingly high (about 50 mmHg for skin), but were confirmed with reflectance spectroscopy. In the volunteer part of the study, the skin measurements were repeated and revealed comparable results to those from rat skin, demonstrating the feasibility of the non-invasive technology to assess mitochondrial function for use in humans.

Taken together, with the help of new technologies as described above, researchers (and possibly in the near future also clinicians) do no longer have to rely on macro-hemodynamic measurements only when it comes to evaluating both the microcirculation and tissue oxygenation, pending that these technologies find their way and acceptance in clinical practice.
